# Accelerated phosphorus recovery from aqueous solution onto decorated sewage sludge carbon

**DOI:** 10.1038/s41598-018-31750-6

**Published:** 2018-09-07

**Authors:** Lingjun Kong, Xingliang Hu, Ziying Xie, Xinyong Ren, Jianyou Long, Minhua Su, Zenghui Diao, Diyun Chen, Kaimin Shih, Li’an Hou

**Affiliations:** 1 Guangdong Provincial Key Laboratory of Radioactive Contamination Control and Resources, School of Environmental Science and Engineering, Guangzhou University, Guangzhou, 510006 P.R. China; 2 Department of Civil Engineering, The University of Hong Kong, Pokfulam Road, Hong Kong, P.R. China; 3 School of Environmental Science and Engineering, Zhongkai University of Agriculture and Engineering, Guangzhou, 510225 China

## Abstract

In search of efficient phosphorus resource recovery and pollution remediation should be highly concerned due to the view of phosphorus nonrenewable and eutrophication. This work presented a new insight into conversion of sewage sludge into favorable carbonaceous adsorbent for accelerated removing and recovering phosphorus from aqueous solution, what addressed the issues of phosphorus recovery and pollution remediation as well as sludge disposal. Ca and water hyacinth were evolved to decorate sludge derived carbon. Effect of mass ratio of sludge, water hyacinth and calcium carbonate on the morphologies and adsorption kinetics was investigated. The adsorbents (SW-Ca-112) resulted from sludge in the presence of water hyacinth and CaCO_3_ in a mass ratio of 1:1:2 had the highest adsorption capacity of 49.50 mg/g P and adsorption rate. Decoration of Ca favored adsorption ability and the presence of water hyacinth accelerated the adsorption rate due to the enhanced porosity. Formation of acicular Ca_5_(PO_4_)_3_OH nanoparticles contributed to the favorable adsorption process. Thus, the contribution of decorated Ca and water hyacinth to the adsorption ability and rate to phosphorus was understand, providing important information on resource utilization of sewage sludge as efficient adsorbent for immobilizing phosphorus from aqueous solution.

## Introduction

Phosphorus (P), as one of raw materials in fertilizer production, is urgently demand in these days. However, the P resource is non-renewable^1^. It is estimated that phosphate rock lasts for only 100 years^2^. Unfortunately, high level of P was founded in some surface water bodies in China due to discharge of P carried by domestic and industrial sewers. The severe runoff of excessive P triggers eutrophication. Resource of P from P pollution water body follows the point view of resource cycle and sustainable, what is prospective. In the past decades, various adsorbents from carbon to minerals were designed to extract P from aqueous solution^3–9^. It is promise to design environmental friendly materials from waste.

Sewage sludge, as an inevitable byproduct generated from sewage treatment, is produced in a large amount around the world as the rapid development of urbanization^10^. Reuse of waste sludge to product is compliant for life cycle assessment studies^11^. It is reviewed from literatures that sludge substances and its derived carbonaceous were widely applied in removing organic compounds, heavy metals^12–14^. Carbonization of sludge to produce low-cost sludge carbon has emerged as a sustainable strategy for sewage sludge management^15^, because this strategy takes the advantages in not only reducing sewage sludge volume but also converting solid waste into adsorbents^13,16–19^ and catalysts^20–24^ for environmental pollution remediation. Extraction of P by sewage sludge and its ash was paid more attention^25^. Recently, Ca-decorated sludge biochar was prepared for phosphorus pollution remediation, in which the sludge biochar could be acted as a carrier and Ca could react with phosphorus by complexation^26^.

However, the surface area is limited comparing to the activated carbon obtained from coal and agriculture waste due to their high mineral contents^27,28^. Addition of carbon rich biomass to sludge could address the above issue^29^. Wu *et al*. reported that the surface area could be increased from 287 to 591 m^2^/g, further increased the microporosity from 5% to 48% due to the addition of biomass^30^.

Water hyacinth (W) is a water weed in several water bodies (e.g., lakers, rivers, canals) around the world^31^. This plant grows rapidly and can completely cover water bodies, causing difficulty in navigation and depletion of nutrients and dissolved oxygen that are essential for aquatic life. These effects have negative impact on the ecologic environment, human health, and economic development^32^. Treatment of water hyacinth has become a serious environmental problem in many parts of the world. Therefore, water hyacinth is considered to be a suitable and an easily available biomass^33^. Considering its high volatile content, utilization of water hyacinth for preparing biochar with loose structure could be considered as one of the most economical and environmental friendly ways for treatment and final disposal of water hyacinth.

In this study, another kind of sewage sludge was resourced as carbonaceous adsorbent for removing and recovering phosphorus, in which it was decorated by Ca, and water hyacinth was added to accelerate its adsorption rate. Herein, water hyacinth was mixed with sewage sludge in varied mass ratio, being carbonized at 800 °C for preparing efficient adsorbent. Batch adsorption experiments were conducted to investigate the adsorption behavior while XRD and SEM techniques were conducted to further understand the adsorption mechanism by morphology and phase analysis. This work gives a new insight into understanding the effect of Ca and water hyacinth decoration on the adsorption capacity and rate to phosphorus from aqueous solution, providing a new view in sludge resource utilization as well as phosphorus removal and recovery.

## Results and Discussion

### Morphology of the biochar

**Figure 1:**
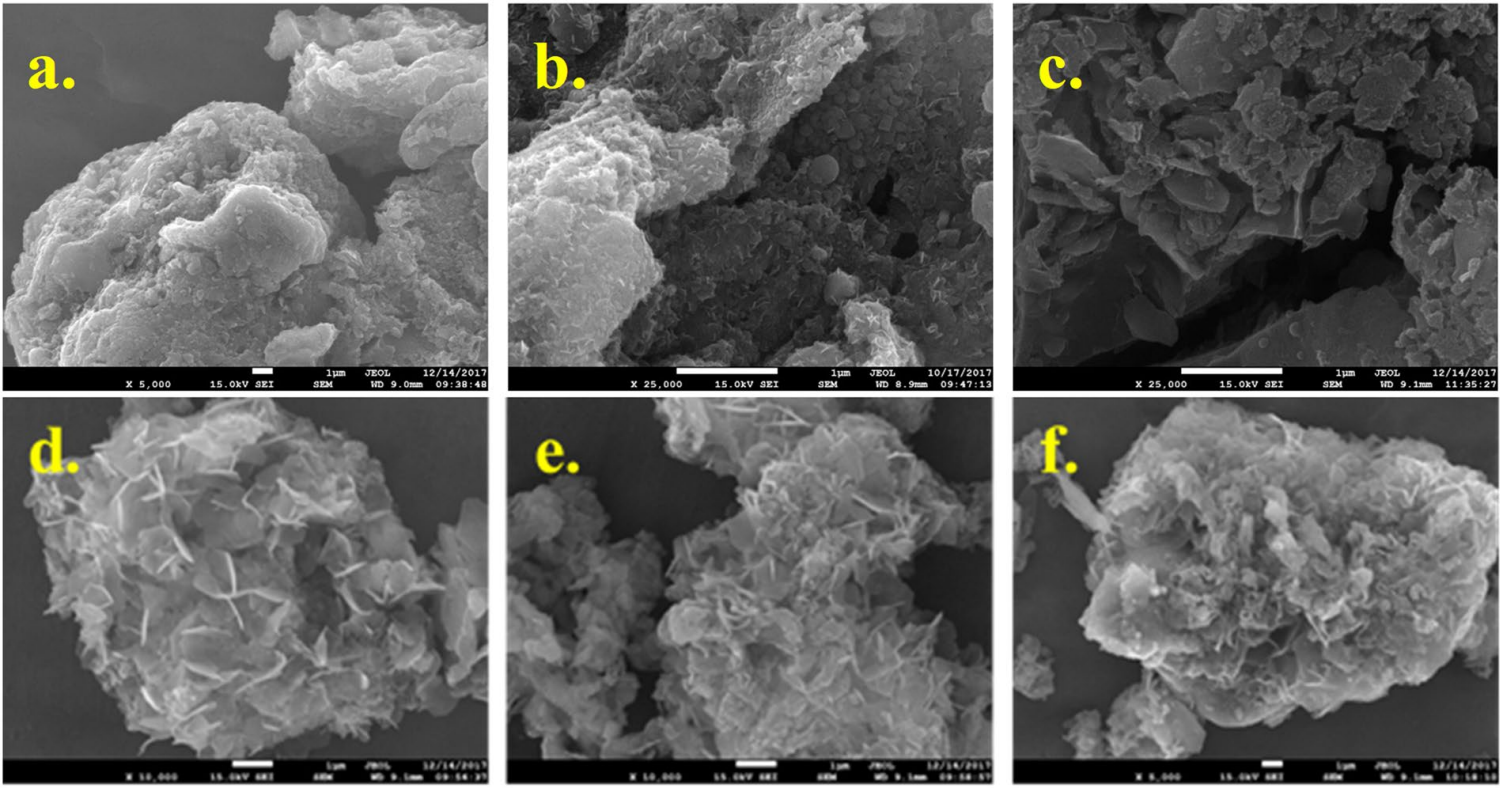
SEM morphologies of the sludge derived biochars: (**a**) sludge char (S), (**b**) S-Ca-11, (**c**) S-W-11, (**d**) SW-Ca-112, (**e**) SW-Ca-325, (f) SW-Ca-415.

Figure 1 presented the micro-morphology of the resulted biochars. Obviously, the particles were agglomerated and tight after being carbonized. Interestingly, some small nanoflakes were observed after addition of CaCO_3_ as shown in Fig. 1(b) comparing to the samples derived from sludge as shown in Fig. 1(a). However, nanoflake was not observed, but many cracks were observed for biochar in the presence of water hyacinth as shown in Fig. 1(c). Amazingly, many cracks and flakes were observed after adding water hyacinth and CaCO_3_ as shown in Fig. 1(d–f). These cracks fabricated due to the addition of water hyacinth and CaCO_3_ could increase the porosity of biochar^30^. The nanoflake was assigned to the Ca derived material. It is different from our previous results that nano-rods were observed in the presence of CaCO_3_^26^. The difference may be due to the interaction between CaCO_3_ and sludge, what could be confirmed in the future. Also, we can observe that the nano-flake structure was different from the ratio of sludge to water hyacinth. The nanoflakes were distinct for the resulted samples in a higher mass ratio of hyacinth to sludge as shown in Fig. 1(d). With the decrease in mass ratio of water hyacinth to sludge, the agglomeration of nanoparticle was observed as shown in Fig. 1(e,f). Because volatile content of water hyacinth was 77.47%, a lot of volatile was decomposed and emitted after carbonization. Thus, the decomposition of volatiles leaded to formation of many cracks and increase in porosity. This result suggested that the presence of water hyacinth increased the porosity of biochar, further providing considerable channels favoring phosphorus adsorption.

### Adsorption behaviors

**Figure 2:**
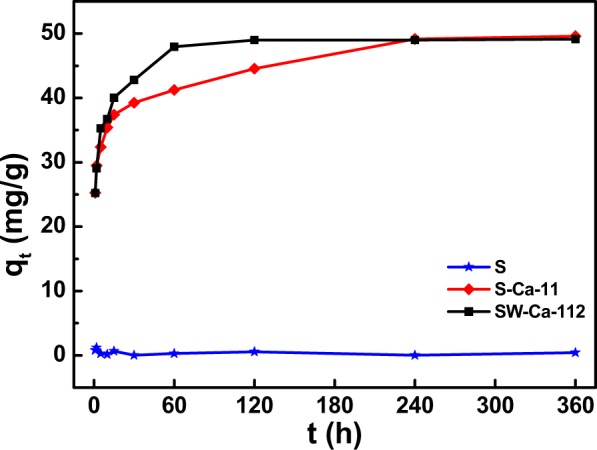
Adsorption capacities of sludge derived char as a function of contact time.

Figure 2 presented the adsorption amounts of phosphorus as a function of contact time. Clearly, the adsorption capacity of sludge char (S) is very low, which is not higher than 5 mg/g. Interestingly, the adsorption capacity highly increased to almost 50 mg/g in the presence of calcium decoration. Adsorption of phosphorus on S could be neglectful, while the addition of CaCO_3_ highly favored phosphorus adsorption, meaning that the favorable adsorption abilities of SW-Ca-112 and S-Ca-11 to phosphorus were ascribed to the decorated calcium. It also can be seen that the adsorption capacities were CaCO_3_ dependent since S-Ca-11 had similar adsorption capacity to SW-Ca-112. Interestingly, the adsorption kinetic process is quite different due to the addition of water hyacinth.

Adsorption kinetic models described in Eqs (2) and (3) were conducted to investigate the kinetic parameters, what were listed in Table 1. Clearly, the pseudo-second-order model fitted well to the adsorption data considering the high correlation coefficients as shown in Table 1. In addition, it can be seen from Table 1 that the adsorption rate of SW-Ca-112 highly increased to 0.0083 in the presence of water hyacinth. It was 2.3 times comparing to that of S-Ca-11 in the absence of water hyacinth, indicating the accelerated adsorption kinetic rate. The increased adsorption rate was agreement with the formed cracks and flakes, suggesting that the cracks and flakes fabricated from decomposition of water hyacinth favored the preferable adsorption rate.

**Table 1:** Adsorption kinetic parameters of phosphorus onto sludge derived adsorbents in the presence of water hyacinth and Ca.

Samples	Pseudo-first-order model			Pseudo-second-order model		
	q_e_ (mg/g)	k_1_ (min^−1^)	R^2^	q_e_ (mg/g)	k_2_ (g/(mg·min))	R^2^
S-Ca-11	17.8678	0.01148	0.8446	49.9500	0.0036	0.9983
SW-Ca-112	17.9382	0.03094	0.9454	49.5050	0.0083	0.9999

**Figure 3:**
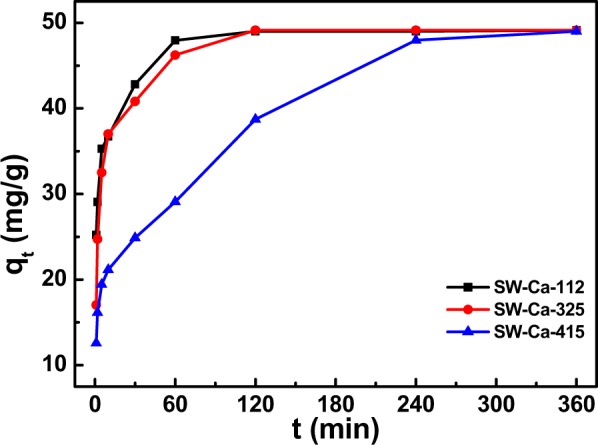
Effect of water hyacinth contents on adsorption capacities as a function of contact time.

To further investigate the effect of addition of water hyacinth on the adsorption behavior, mass content of CaCO_3_ was fixed to 50%, and the adsorption capacities of adsorbents in varied mass ratio of sludge to water hyacinth were presented in Fig. 3. They had the same equilibrium adsorption capacities of about 49.50 mg/g P, but the reaction time to equilibrium was different. SW-Ca-112, SW-Ca-325 had similar equilibrium time of 120 min because the mass ratio of sludge to water hyacinth was similar, while SW-Ca-415 had a longer equilibrium time of 240 min due to the relative low water hyacinth content. Also, as can be seen from Fig. 1(f), the cracks of SW-Ca-415 were not as distinct as those of SW-Ca-112 and SW-Ca-325. Therefore, the shorter time to equilibrium of SW-Ca-112 and SW-Ca-325 than that of SW-Ca-415 was concluded from the fabricated cracks by decomposition of water hyacinth. The presence of water hyacinth highly decreases the time to equilibrium. In addition, it is not hard to understand that the adsorption capacities are determined by the decorated Ca as shown in our previous study^34^.

**Figure 4:**
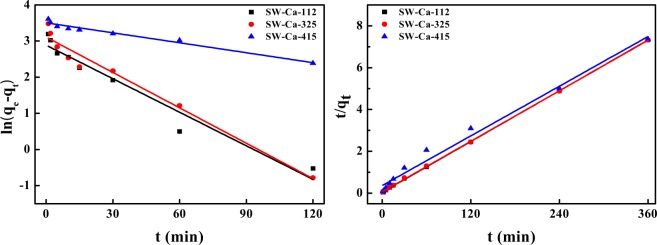
Linear fitted adsorption kinetics of phosphorus on the SW-Ca with varied mass ratio: (**a**) pseudo-first-order model and (**b**) pseudo-second-order model.

Two widely used pseudo-first-order and pseudo-second-order models were presented to fit the above experimental data to evaluate the adsorption rate, and the nonlinear fitted technique was applied to estimate the kinetic parameters^35^. However, the nonlinear optimization was not fit for the experimental data adequately (Fig. S1). Linear optimization techniques were applied to estimate the experimental results and corresponding parameters were listed in Fig. 4 and Table 2. Considering the correlation coefficients shown in Table 2, adsorption of P onto SW-Ca-112, SW-Ca-325 fitted well by the pseudo-second-order model. In addition, the fitted equilibrium adsorption capacities of 49.50 and 49.68 mg/g onto SW-Ca-112, SW-Ca-325 were quite agreement with the experimental adsorption capacities. Since the pseudo-second-order model is based on the assumption that adsorption of P onto adsorbent involves the chemical reaction between PO_4_^3−^ and Ca, as reported by Mitrogiannis *et al*.^36^, indicating a chemisorptions-dominated process. Also, the adsorption capacity to P is higher than the previous results^36–39^. Importantly, the fitted adsorption rate calculated from pseudo-second-order model for SW-Ca-112 was higher than that for SW-Ca-325 due to higher content of water hyacinth. In addition, the SW-Ca-415 had quite low adsorption rate due to the low content of water hyacinth. These results were quite agreement with the fabricated cracks of SW-Ca-112, SW-Ca-325 and SW-Ca-415 as shown in Fig. 1. Thus, these results all confirmed that the presence of water hyacinth could accelerate the adsorption rate due to fabrication of favorable cracks.

**Table 2:** Adsorption kinetic parameters of phosphorus onto SW-Ca derived adsorbents with varied water hyacinth and sludge content.

Samples	Pseudo-first-order model			Pseudo-second-order model		
	q_e_ (mg/g)	k_1_ (min^−1^)	R^2^	q_e_ (mg/g)	k_2_ (g/(mg·min))	R^2^
SW-Ca-112	17.9382	0.0309	0.9454	49.5050	0.0083	0.9999
SW-Ca-325	22.4502	0.0326	0.9656	49.6771	0.0062	0.9998
SW-Ca-415	33.2908	0.0092	0.9672	50.5306	0.0011	0.9852

**Figure 5:**
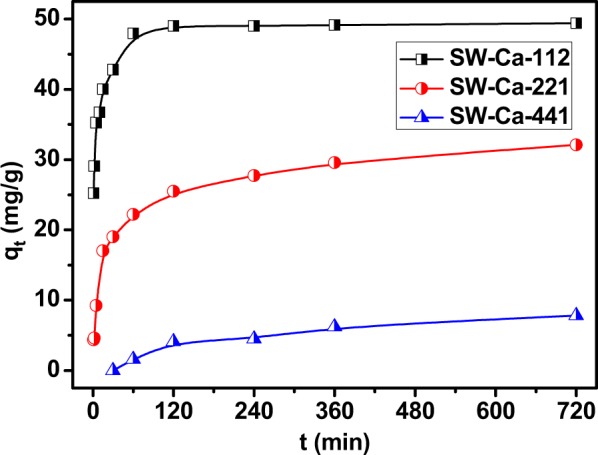
Effect of CaCO_3_ contents on the P adsorption capacity.

Figure 5 showed that the adsorption capacity of P depended on the CaCO_3_ content. Pseudo-first-order and pseudo-second-order models were performed to fit the experiment data. The fitted parameters were listed in Table 3. Clearly, pseudo-second-order model was better than pseudo-first-order model to fit the adsorption experiments as indicated by the favorable correlation coefficients. In addition, the calculated equilibrium adsorption capacities for SW-Ca-112, SW-Ca-221 and SW-Ca-441 were quite agreement with the experimental data. The SW-Ca-441 with the lowest CaCO_3_ content had the lowest adsorption capacity to P. This result confirmed that the favorable adsorption capacity to P was determined by the chemical reaction of P with Ca. In addition, the adsorption rates calculated from pseudo-second-order model were agreement with the CaCO_3_ ratio. The higher CaCO_3_ ratio, the higher adsorption rate was. Thus, it is easy to conclude that the decorated Ca plays a key role in influencing the adsorption capacity.

**Table 3:** Adsorption kinetic parameters of phosphorus onto SW-Ca derived adsorbents with varied Ca content.

Samples	Pseudo-first-order model			Pseudo-second-order model		
	q_e_ (mg/g)	k_1_ (min^−1^)	R^2^	q_e_ (mg/g)	k_2_ (g/(mg⋅min))	R^2^
SW-Ca-112	18.2910	2.0467	0.9620	49.1642	0.7410	0.9999
SW-Ca-221	23.8566	0.6490	0.8303	33.3222	0.07340	0.9989
SW-Ca-441	10.9412	0.4155	0.9994	10.6168	0.0204	0.9864

### Characterization of adsorption products

**Figure 6:**
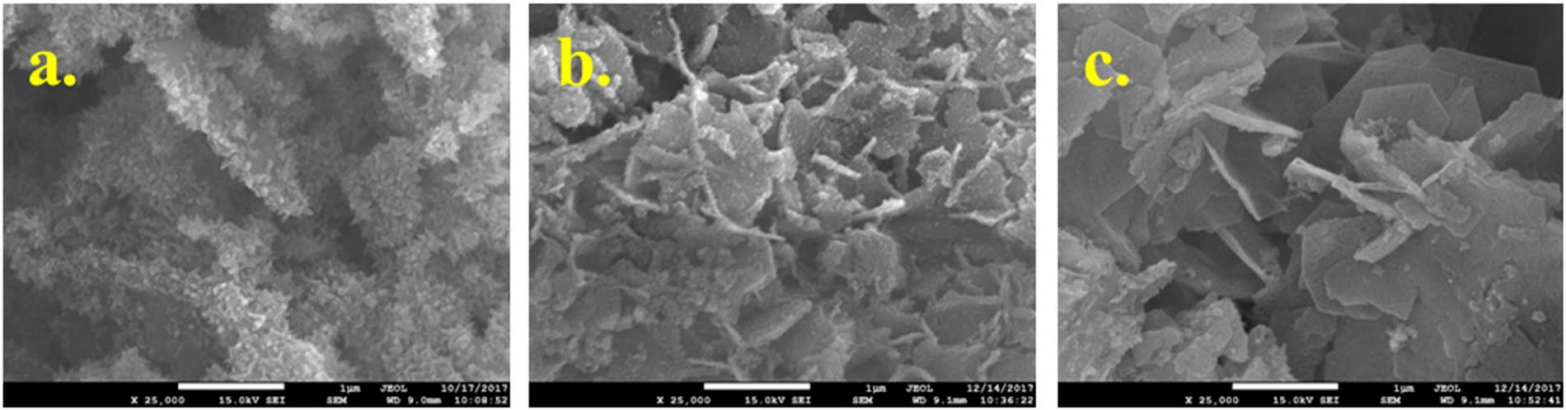
SEM morphologies of (**a**) SW-Ca-112, (**b**) SW-Ca-325 and (**c**) SW-Ca-415 after adsorption of P.

The products after adsorption of P were characterized by SEM analysis as shown in Fig. 6. Comparing to the samples before adsorption, nanoparticles in acicular was observed on the nanoflakes. Especially, these nanoparticles were well dispersed onto the well dispersed nanoflakes of SW-Ca-112 (as shown in Fig. 6(a)). For the other SW-Ca-325 and SW-Ca-415, nanoparticles were also observed on the surface of nanoflakes. The observed nanoparticles could be ascribed to the crystal products after reaction of P with Ca.

**Figure 7:**
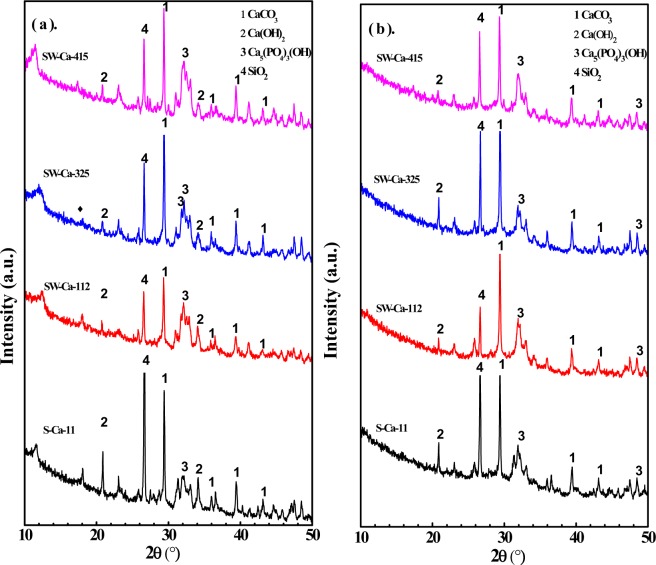
XRD patterns of the sludge derived biochars in the presence of Ca and water hyacinth (**a**) before adsorption, (**b**) after adsorption.

XRD analysis of the adsorbents before and after phosphorus adsorption was conducted in Fig. 7 to understand the phase of the crystal nanoparticles after adsorption. The diffraction peaks assigned to CaCO_3_ and Ca(OH)_2_ were observed in the presence of CaCO_3_. The presented Ca(OH)_2_ was due to the decomposition of CaCO_3_ into CaO and CO_2_, further the CaO can reacted with the water during adsorption to form Ca(OH)_2_^26^. Besides, diffraction peaks located at 33.2°, being assigned to Ca_5_(PO_4_)_3_OH, were both observed for adsorbents before and after P adsorption. The presence of Ca_5_(PO_4_)_3_OH before P adsorption was due to the initial P content in these sludge derived chars were about 1.17 to 1.71% (Table S2). And Ca_5_(PO_4_)_3_OH crystal was also observed after P adsorption, but mass content of P after adsorption increased to 8.46% (Table 4). The increase in the P content indicated favorable P adsorption, and the Ca_5_(PO_4_)_3_OH crystal was the fate of P after adsorption.

**Table 4:** EDS analysis results (wt%) of the sludge derived biochars in the presence of various ratios of sludge to Ca and water hyacinth after P adsorption.

Sample	C	O	Mg	Al	Si	P	K	Ca	Fe
SW-Ca-112	28.84	41.01	0.66	0.68	2.49	5.91	0.26	18.9	1.25
SW-Ca-415	11.52	45.51	0.6	1.41	3.03	6.93	0.28	28.73	1.98
SW-Ca-325	15.57	44.82	0.62	1.04	2.64	8.46	0.4	24.58	1.88
S-Ca-11	14.81	44.46	0.46	1.76	4.24	7.62	0.52	23.93	2.2

## Conclusion

Sewage sludge was successfully converted into carbonaceous adsorbent for removing and recovering phosphorus from aqueous solution, in which decorated Ca favored adsorption capacity while added water hyacinth accelerated the adsorption rate. Nanoflakes were observed due to Ca decoration, and carbonization of water hyacinth leaded to the occurrence of cracks, contributing to the increase in adsorption capacity and rate. Pseudo-second-order model fitted well to the adsorption process. Formation of acicular Ca_5_(PO_4_)_3_OH nanoparticles contributed to the favorable adsorption process. Therefore, this work provides a new insight into conversion of sludge and water hyacinth into adsorbent in application of phosphorus pollution remediation.

## Materials and Methods

### Materials

Water hyacinth (W) was collected from the Pear River in Guangzhou, further being dried and ground into powder less than 100 meshes. The volatile content was 77.47%. Dewatered sewage sludge was collected from Lijiao sewage treatment plant, in Guangzhou, China, and subsequently being dried by an oven set at 105 °C. Table S1 showed that the volatile of dried sludge was 31.47%. C. O, Al, Si, Fe were the main elements in dried sludge. Also, Ca and P were also observed in the initial dried sludge. The ash mass content of the dried sludge is high to 48.54%. Calcium carbonate (CaCO_3_) and potassium phosphate (K_3_PO_4_) were chemical grade purchased from sigma reagent Corporation, USA.

### Preparation of adsorbent

Ca-decorated biochar was prepared by carbonization in a programmable tube furnace at 800 °C. Firstly, the sludge was mixed with CaCO_3_ and water hyacinth in a mass ratio of 1:1, being carbonized at 800 °C in a tube furnace (SKF-210, Hangzhou Lantian Instrument Co., China) and washed by deionized water to remove the residual ions. The obtained sample was named as S-Ca-11 according to the mass ratio of sludge to CaCO_3_ was 1:1. In addition, the sludge, water hyacinth and CaCO_3_ were mixed in varied mass ratio to investigate the effect of each component on the adsorption behavior. These samples were prepared as described above. These resulted samples were named as SW-Ca-112, SW-Ca-325, SW-Ca-415, SW-Ca-221, SW-Ca-441 according to the mass ratio of sludge, water hyacinth and CaCO_3_ were1:1:2, 3:2:5, 4:1:5, 2:2:1, 4:4:1, respectively.

### Adsorption experiments

A stock solution containing 1,000 mg/L P was prepared by dissolving K_3_PO_4_ in deionized water, and the desired solutions were prepared by diluting the stock solution. In the adsorption experiments, the adsorbent dose was 1.000 g/L. All concentrations are expressed in P-PO_4_^3−^. All batch adsorption experiments were performed in conical flasks with a plug on a shaker equipped with a thermostat at 200 rpm and at 25 °C.

Adsorption kinetic was investigated by conducting an initial P concentration of 50 mg/L, and the residual sample at each determined time interval was drawn. For each test, the suspension was filtrated through a 0.45 μm cellulose acetate membrane, and the residual P concentration in the filtrate was measured using an ultraviolet spectrophotometer at 700 nm (HITACHI U-2910, Japan). The adsorption amount was calculated as the difference between the initial and residual concentrations.

All experiments were repeated thrice, and the average value was calculated. The adsorption capacities at different time t (q_t_, mg/g) were calculated as follows:

1 $${q}_{t}=\frac{({C}_{0}-{C}_{t})V}{m}$$

where C_0_ and C_t_ (mg/L) represent the P concentration at initial and t time (min), respectively. V (L) represents the volume of the solution, and m (g) represents the adsorbent mass.

### Adsorption kinetic models

To further understand the effect of the addition of Ca and water hyacinth on the adsorption behavior of P, two widely used pseudo-first-order and pseudo-second-order kinetic models were linear fitted as shown below to describe the adsorption kinetics^40^.

2 $$\mathrm{ln}({q}_{e}-{q}_{t})=\,\mathrm{ln}\,{q}_{e}-{k}_{1}t$$

3 $$\frac{t}{qt}=\frac{1}{{k}_{2}{{q}_{e}}^{2}}+\frac{t}{{q}_{e}}$$

where q_t_ and q_e_ (mg/g) represented the adsorption amounts of phosphorus at t time and equilibrium, respectively. t (min) represents the adsorption time. k_1_, k_2_ were the adsorption rate constants of pseudo-first-order model, pseudo-second-order kinetic model, respectively.

### Analytical method

The major phase of biochar before and after adsorption of PO_4_^3−^ were characterized through XRD analysis using a D/max 2200 vpc diffractometer (Rigaku Corporation, Japan) with Cu Kα radiation at 40 kV and 30 mA, to investigate their transformation behaviors and fates. Scanning electron microscopy (SEM) images were observed using a JEOL JSM-6330F-mode Field Emission Scanning Electron Microscope (JED-2300).

## Electronic supplementary material

Supporting information
