# Supplementary material for: Accelerated phosphorus recovery from aqueous solution onto decorated sewage sludge carbon
Source: Sci Rep. 2018 Sep 7;8:13421. doi: 10.1038/s41598-018-31750-6 (PMC6128935; doi:10.1038/s41598-018-31750-6)
Supplement: Supplementary file 1 — Supporting information [file 41598_2018_31750_MOESM1_ESM.docx]

**Accelerated phosphorus recovery from aqueous solution onto decorated sewage sludge carbon**

Lingjun Kong^1^^,2*^, Xingliang Hu^1^, Ziying Xie^1^, Xinyong Ren^1^, Jianyou Long^1^, Minhua Su^1^, Zenghui Diao^3^, Diyun Chen^1^, Kaimin Shih^2^ Li’an Hou^1^

^1^ Guangdong Provincial Key Laboratory of Radioactive Contamination Control and Resources, School of Environmental Science and Engineering, Guangzhou University, Guangzhou, 510006, P. R. China

^2^ Department of Civil Engineering, The University of Hong Kong, Pokfulam Road, Hong Kong, P. R. China

^3^ School of Environmental Science and Engineering, Zhongkai University of Agriculture and Engineering, Guangzhou 510225, China.

^*^ Corresponding author (s): [kongl_jun@163.com](mailto:kongl_jun@163.com) (LJ Kong).

Table S1 Proximate and EDS elemental analysis of dried sludge and water hyacinth.

| Volatile | Ash | EDS element content (wt. %) | | | | | | | | |
| --- | --- | --- | --- | --- | --- | --- | --- | --- | --- | --- |
|  |  | C | O | Mg | Al | Si | P | K | Ca | Fe |
| 31.47 | 48.54 | 21.26 | 58.44 | 0.75 | 3.78 | 8.08 | 1.93 | 0.61 | 1.82 | 3.23 |

Table S2 EDS analysis results (wt. %) of the sludge derived biochars in the presence of various ratios of sludge to Ca and water hyacinth.

| Sample | C | O | Mg | Al | Si | P | K | Ca | Fe |
| --- | --- | --- | --- | --- | --- | --- | --- | --- | --- |
| SW-Ca-112 | 30.66 | 41.95 | 0.42 | 2.39 | 3.97 | 1.17 | 0.4 | 16.97 | 2.06 |
| SW-Ca-415 | 10.29 | 46.01 | 0.49 | 3.27 | 5.34 | 1.61 | 0.25 | 30.47 | 2.27 |
| SW-Ca-325 | 15.87 | 45.22 | 0.67 | 2.23 | 3.71 | 1.71 | 0.21 | 28.56 | 1.82 |
| S-Ca-11 | 17.65 | 47.42 | 0.61 | 3.15 | 6.55 | 1.57 | 0.49 | 19.85 | 2.7 |

Fig. S1 Nonlinear fitted adsorption kinetics of phosphorus.
